# Upper and small bowel Crohn's disease in Brazilian children: Phenotypic characteristic and surgical risk

**DOI:** 10.1002/jpn3.70411

**Published:** 2026-03-26

**Authors:** Jane Oba, Carlos Walter Sobrado, Elizete Aparecida Lomazi, Mariana Deboni, Michela Cynthia da Rocha Marmo, Vanessa Adriana Scheeffer, Silvio da Rocha Carvalho, Adérson Omar Mourão Cintra Damião, Matheus Freitas Cardoso de Azevedo, Carlos Frederico Sparapan Marques, Eytan Wine, Artur Figueiredo Delgado, Clovis Artur Almeida da Silva Full

**Affiliations:** ^1^ Departamento de Gastroenterologia e Coloproctologia, Hospital das Clínicas da Faculdade de Medicina da Universidade de São Paulo, Hospital Israelita Albert Einstein São Paulo Brazil; ^2^ Departamento de Coloproctologia, Hospital das Clínicas da Faculdade de Medicina da Universidade de São Paulo São Paulo Brazil; ^3^ Departamento de Pediatria, Faculdade de Ciências Médicas da Universidade Estadual de Campinas Campinas Brazil; ^4^ Unidade de Gastroenterologia Pediátrica, Instituto da Criança e do, Adolescente da Faculdade de Medicina da Universidade de São Paulo São Paulo Brazil; ^5^ Departamento de Pediatria Universidade Federal de Pernambuco Recife Brazil; ^6^ Serviço de Gastroenterologia pediátrica, Santa Casa de Misericórdia de, Porto Alegre Porto Alegre Brazil; ^7^ Serviço de Gastroenterologia Pediátrica Universidade Federal do Rio, de Janeiro Rio de Janeiro Brazil; ^8^ Departamento de Gastroenterologia, Hospital das Clínicas da Faculdade de Medicina da Universidade de São Paulo São Paulo Brazil; ^9^ Disciplina de Coloproctologia, Hospital das Clínicas da Faculdade de, Medicina Universidade de São Paulo São Paulo Brazil; ^10^ Division of Gastroenterology, Hepatology and Nutrition Hospital for Sick Children Toronto Ontario Canada; ^11^ Departamento de Pediatria Instituto da Criança e do Adolescente, Hospital das Clínicas da Faculdade de Medicina da Universidade de São Paulo São Paulo Brazil

**Keywords:** pediatric inflammatory bowel disease, penetrating disease, stricturing disease, stricturoplasty

## Abstract

**Objectives:**

Upper and small bowel Crohn's disease (U‐SBCD) represents a clinically aggressive phenotype with high complication rates yet remains diagnostically challenging. In low‐ and middle‐income countries (LMICs), limited inflammatory bowel disease (IBD) awareness contributes to diagnostic delays, but their impact on U‐SBCD outcomes remains poorly characterized. We aimed to characterize clinical features, management patterns, and surgical outcomes of pediatric U‐SBCD in Brazil, establishing IBD baseline for future regional comparisons in LMIC setting.

**Methods:**

This multicenter retrospective study analyzed 124 pediatric Crohn′s disease (CD) patients (diagnosed < 18 years) from six Brazilian centers according to revised PORTO‐European Society for Pediatric Gastroenterology, Hepatology, and Nutrition (ESPGHAN) criteria. Patients with U‐SBCD (Paris L4a/L4b, *n* = 39) were compared to non‐SBCD (L1/L2/L3). Univariate and multivariable analyses assessed demographics, diagnostic delay, disease behavior, growth impairment, and therapeutic outcomes over 18 months follow‐up. The population comprised predominantly African–Brazilian admixture.

**Results:**

U‐SBCD patients presented at older age (median 14 vs. 12 years; *p* = 0.001) with greater height impairment (*p* = 0.010) and lower body mass index (BMI)/age *z*‐scores (−1.14 vs. −0.44; *p* = 0.029). Severe weight loss (*z*‐score < −2) conferred 8.3‐fold increased U‐SBCD odds (95% confidence interval [CI]: 2.17–31.66; *p* = 0.002). Each year of diagnostic delay increased U‐SBCD odds by 22% (95% CI: 1.06–1.39; *p* = 0.004). U‐SBCD patients required more biologic therapy (97% vs. 75%; *p* = 0.003) with higher treatment escalation rates (31.6% vs. 21.2%; *p* = 0.004). Multivariate analysis confirmed U‐SBCD as an independent three‐fold predictor of surgery (OR 3.02; 95% CI: 1.02–8.94; *p* = 0.046). A 9‐year‐old girl with U‐SBCD died perioperatively. Psychiatric symptoms affected 30% of both groups.

**Conclusion:**

In LMIC settings, strengthening medical education is essential to improve early recognition of U‐SBCD and prevent progression to irreversible complications. Notably, the phenotypic pattern observed in our study more closely resembled that reported in Asian pediatric patients than Caucasian cohorts.

## Abstract (highlights)

**What is Known:**

- Pediatric upper and small bowel Crohn′s disease (U‐SBCD) is an aggressive phenotype that often presents with mild symptoms but carries a risk of strictures and surgeries due to its significant inflammatory burden.

**What is New:**

- This study provides the first large‐scale, multicenter characterization of pediatric U‐SBCD in Brazil, establishing baseline data in low‐ and middle‐income countries settings.
- Each year of diagnostic delay tripled surgical risk, highlighting delay as a preventable cause of adverse outcomes.
- Brazil′s admixed population, with predominantly African ethnicity, U‐SBCD phenotype more closely resembled Asian than Caucasian cohorts, raising questions about biological mechanisms of disease expression.

## Graphical abstract

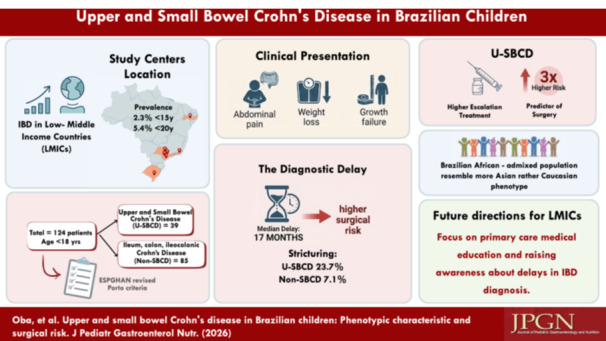

## 1 INTRODUCTION

Crohn′s disease (CD) is a chronic and progressive inflammatory bowel disease (IBD) with rising global incidence, particularly in low‐ and middle‐income countries (LMICs) across Latin America, Asia, and Africa.^1^, ^2^, ^3^ In Brazil, pediatric IBD prevalence is increasing rapidly; however, diagnosis rates before 15 years (2.3%) and before 20 years (5.4%) with approximately half as CD, remain substantially lower than international reports. These figures likely reflect diagnostic delays or missed cases rather than lower true incidence.^4^ Pediatric‐onset CD often presents with aggressive phenotypes, and complications such as growth impairment, pubertal delay, and reduced quality of life, highlighting the urgent need for early diagnosis and optimized therapy.^5^, ^6^ These challenges are particularly pronounced in LMICs settings.^7^

Although CD has been treated as a single entity, small bowel and colonic CD represent biologically and clinically distinct subtypes with different pathogenic mechanisms and therapeutic responses.^8^ Compared to colonic disease, small bowel CD is strongly associated with Nucleotide‐binding oligomerization domain‐containing protein 2 (NOD2) variants, decreased bacterial diversity, and higher risk of stricturing/penetrating phenotypes and surgery.^1^, ^5^, ^8^ Within the Paris Classification, upper and small bowel involvement (L4a: proximal to ligament of Treitz; L4b: distal to ligament of Treitz but proximal to distal third of ileum), collectively termed here as U‐SBCD, represents a distinct phenotype from disease limited to terminal ileum (L1), colon (L2), or ileocolonic (L3), termed here as non‐SBCD.^9^ This distinction is clinically critical because pediatric U‐SBCD represents a high‐risk phenotype associated with severe disease course.^5^, ^8^, ^10^, ^11^

Diagnostic delay by years remains a key challenge of U‐SBCD due to non‐specific symptoms, often mistaken for functional disorders or infections like tuberculosis.^12^, ^13^ By presentation, many children already have stricturing (B2) or penetrating (B3) complications that contribute to growth failure, early surgical resections.^5^

Despite its aggressive nature, exclusive pediatric U‐SBCD data rely on extrapolated from adult cohorts or mixed pediatric‐adult analyses.^14^ This knowledge gap is pronounced in Brazil and other LMICs, where IBD awareness remains poorly integrated into medical education, and limited access to specialized small bowel imaging contributes to diagnostic delays.^7^, ^15^ To address the lack of robust data, we conducted a multicenter characterization of pediatric U‐SBCD in Brazil. Our primary objective was to characterize clinical presentation, diagnostic features, treatment patterns, and surgical outcomes between U‐SBCD and non‐SBCD phenotypes. Secondary objectives included quantifying diagnostic delay, identifying factors associated with complications, evaluating fecal calprotectin (FC) utility, and establishing baseline data for future LMIC comparisons.

## 2 METHODS

### 2.1 Ethics statement

The study was approved by the Ethics Committee of São Paulo University Hospital (CAAE: 82679518.2.0000), and informed consent was obtained from legal guardians and participating patients. For participants under 18 years, forms were signed by legal guardians with adolescent assent. For patients over 18 years, diagnosed in childhood, consent was obtained directly.

### 2.2 Patients and study design

This multicenter retrospective study analyzed patient records from six Brazilian gastroenterological centers specialized in pediatric and adult IBD treatment, conducted between January 2023 and July 2024. All centers provided standardized diagnostic and therapeutic protocols aligned with CD diagnosis established according to modified Porto criteria.^16^

### 2.3 Phenotypic classification

This study enrolled 124 children and adolescents with CD, categorized into two groups based on macroscopic disease location at diagnosis according to the Paris classification,^9^ providing phenotypic stratification. Patients were classified at baseline after diagnostic workup. Phenotypic evolution was assessed during follow‐up, and patients were reclassified if disease behavior progressed (B1 to B2/B3) or if they underwent CD‐related abdominal surgery.

#### 2.3.1 U‐SBCD group

Disease with proximal small bowel involvement: L4a: Disease proximal to the ligament of Treitz; L4b: Disease distal to the ligament of Treitz but proximal to the distal third of the ileum. Patients could have variable segmental distribution. The classification was determined by primary location.^9^

#### 2.3.2 Non‐SBCD group

Disease limited to terminal ileum (L1), colon (L2), or ileocolonic region (L3). Patients could have variable segmental distribution distal ileum‐colon.

#### 2.3.3 Inclusion criteria

Patients were included if they: (a) received CD diagnosis before 18 years of age; (b) had complete diagnostic workup including upper endoscopy, ileocolonoscopy, cross‐sectional imaging (CTE or MRE), and histology (Porto criteria).^16^

Patients were classified at baseline after diagnostic workup. Phenotypic evolution was assessed during follow‐up, and patients were reclassified if disease behavior progressed (B1 to B2/B3) or if they underwent CD‐related abdominal surgery.

#### 2.3.4 Exclusion criteria

Behçet′s disease, vascular lesions, NSAID‐induced enteropathy, peri‐appendiceal cecal abscess, tubo‐ovarian disease, intestinal infections or parasitic agents, ulcerative colitis, IBD‐unclassified, and malignancy. All patients were screened for tuberculosis.

#### 2.3.5 Perianal disease

This was categorized as phenotypic behavior modifier (p), excluding minor manifestations such as isolated violaceous skin tags, simple fissure or hemorrhoids. Only active fistulae and/or abscesses requiring intervention met criteria.^9^

### 2.4 Clinical and laboratory assessment

#### 2.4.1 Demographic and anthropometric data

Ethnicity (self‐report), sex, and age at diagnosis were recorded. Body mass index (BMI)/age *z*‐scores and Height for age *z*‐score were calculated using WHO Anthro/WHO AntroPlus, with malnutrition defined as *z* < −2.^17^ Growth impairment (G1) was defined according to Paris classification.^9^

#### 2.4.2 Laboratory evaluation

Comprehensive assessment at diagnosis included: Complete blood count; liver and pancreas biochemistry (alanine transaminase, aspartate transaminase, gamma‐glutamyl transferase, alkaline phosphatase, bilirubin, amylase, lipase); nutritional markers (albumin, 25‐OH vitamin D₃); inflammatory markers (C‐reactive protein, erythrocyte sedimentation rate [ESR]); FC: measured using ELISA, with normal cutoff <250 μg/g.^18^, ^19^

#### 2.4.3 Diagnostic delay

Duration in months between symptom onset and confirmed CD diagnosis.^12^ Disease activity: Assessed using pediatric Crohn′s disease activity index (PCDAI): remission <10; mild 10–27.5; moderate 30–37.5; severe 40–100.^20^ Clinical symptoms: Abdominal pain, diarrhea (including bloody stools), weight loss, vomiting, and distension were systematically recorded.

#### 2.4.4 Endoscopic evaluation

All patients underwent standardized upper endoscopy and ileocolonoscopy. Direct mucosal examination and histological analysis identified granulomas, excluded infections and malignancies, and confirmed CD diagnosis according to Porto criteria.^16^

CTE/MRE was performed in all patients to assess small bowel involvement to provided full‐thickness wall, strictures, penetrating disease, and extramural complications.^2^ MRE was preferred when available due to lack of radiation exposure and superior soft tissue characterization. Capsule endoscopy and intestinal ultrasonography (IUS) were unavailable at participating centers during the study period.

### 2.5 Treatment

#### 2.5.1 Exclusive enteral nutrition (EEN)

EEN was used for a very few selected inpatients. CD exclusion diet with partial enteral nutrition was not used. Corticosteroids were used for induction. Immunomodulators: Thiopurines and methotrexate were adjuncts to biologics for maintenance therapy. Biologic agents: Infliximab (first‐line), adalimumab (second‐line), vedolizumab (off‐label for refractory cases). Treat‐to‐target strategy was employed with close monitoring according to STRIDE‐II recommendations.^19^ Medication use refers exclusively to initial therapy prescribed within 90 days of diagnosis.

#### 2.5.2 Surgical

History, type, and location of intestinal resections were documented. Specific techniques included stricturoplasty, segmental resection, ileocecal resection, stoma formation, side‐to‐side anastomosis, and Kono‐S anastomosis.^21^ Emergency or elective procedures were included. Anorectal procedures related to perianal disease were cataloged separately. These included anorectal abscess drainage, seton placement, and fistulectomy.

### 2.6 Outcome measures

#### 2.6.1 Primary outcomes

Complicated disease behavior (stricturing B2/penetrating B3), surgical intervention within 18‐month follow‐up.

#### 2.6.2 Secondary outcomes

Treatment response (clinical remission, need for dose escalation/switch), growth impairment, postoperative recurrence. Psychiatric comorbidities were documented when requiring psychiatric referral and treatment.

### 2.7 Statistical analysis

Statistical analysis was performed using IBM‐SPSS v22.0. Quantitative parameters used mean, standard deviation, median, and quartiles, compared via unpaired *t*‐Student or Mann–Whitney tests. Qualitative characteristics were analyzed using absolute/relative frequencies and chi‐square or exact tests. Pearson or Spearman correlations were calculated for *Z*‐score, with its relationship to qualitative variables assessed via unpaired *t*‐Student or analysis of variance. Multivariate logistic regression models were employed to explain the diagnosis and surgical complications, while a multiple linear regression model assessed factors influencing the *Z*‐score with a significance level (*p* ≤ 0.05).

## 3 RESULTS

### 3.1 Study population and baseline characteristics

One hundred twenty‐four pediatric patients with CD met inclusion criteria. Among these, 39 patients (31.5%) had U‐SBCD: 8 (20.5%) with L4a involvement and 31 (79.5%) with L4b disease. The remaining 85 patients (68.5%) presented with non‐SBCD phenotypes (L1/L2/L3).

Table 1 presents baseline demographic and anthropometric characteristics. Median age at diagnosis was significantly higher in U‐SBCD compared to non‐SBCD (14‐years [interquartile range, IQR: 11–17] vs. 12 years [IQR 8.5–14]; *p* = 0.001). Sex distribution was similar (71.1% male in U‐SBCD vs. 58.8% in non‐SBCD; *p* = 0.195). The population exhibited diverse self‐reported ethnicity, predominantly African Brazilian, making standardized racial classification difficult.

**Table 1:** Demographic and baseline anthropometrics characteristics of U‐SBCD patients and non‐SBCD.

Variables	U‐SBCD (*n* = 39)	Non‐SBCD (*n* = 85)	*p*
Age at diagnosis, years	14 (11‐17)	12 (8.5‐14)	0.001^b^
Gender (% male)	27 (71.1)	50 (58.8)	0.195^a^
BMI, kg/m^2^	17 (15–20)	18 (14‐20)	0.724^b^
Height (cm) mean ± SD	156 ± 20.8	143.3 ± 26.1	0.010^b^
Weight (kg) mean ± SD	44.2 ± 16	38.9 ± 17.5	0.116
*z*‐score (BMI/age)mean ± SD	−1.14 ± 1.57	−0.44 ± 1.62	0.029^b^

*Note*: Results are presented in mean (±SD), median (minimum and maximum values), or *n* (%). U‐SBCD: L4a, L4b; non‐SBCD: ileum, colonic ileocolonic CD.

Abbreviations: BMI, body mass index; CD, Crohn′s disease; SD, standard deviation; U‐SBCD, upper and small bowel Crohn′s disease.

a Chi‐square test.

b Student *t*‐test.

### 3.2 Clinical presentation and diagnostic delay

Presenting symptoms differed significantly between phenotypes (Table 2). Abdominal pain was markedly more prevalent in U‐SBCD (78.9% vs. 43.5%; *p* < 0.001), while bloody diarrhea showed no discriminative value (15.8% vs. 23.5%; *p* = 0.331). Weight loss was more frequent in U‐SBCD (63.2% vs. 38.8%; *p* = 0.012), and growth impairment was observed in 42% of U‐SBCD patients compared to 23% of non‐SBCD (*p* = 0.036).

**Table 2:** Patient′s clinical presentation and diagnostic characteristics.

	U‐SBCD (*n* = 39)	Non‐SBCD (*n* = 85)	*p*
Delayed diagnosis (months) mean ± SD	17 ± 15.5	13 ± 10.9	0.191^a^
Initial symptoms			
Abdominal pain (%)	30 (78.9)	37 (43.5)	<0.001
Weight loose (%)	24 (63.2)	33 (38.8)	0.012
Growth impairment (%)	16 (42)	20 (23)	0.036^b^
Diarrhea, *n* (%)	14 (36.8)	39 (45.9)	0.350
Blood diarrhea *n* (%)	6 (15.8)	20 (23.5)	0.331
Behavior, *n* (%)			0.050^c^
inflammatory (B1) (%)	25 (65.8%)	65 (76.4%)	
Stricturing (B2) (%)	9 (23.7%)	6 (7.1%)	
Penetrating (B3) (%)	5 (10,5%)	14 (16.5%)	
Perianal, *n* (%)	7 (18.4%)	31 (36.5%)	0.045
PCDAI moderate and severe, *n* (%)	25 (66%)	27 (32%)	<0.001^d^
Extraintestinal manifestation	8 (21%)	20 (23%)	
Hemoglobin (gm/dL) mean ± SD	11.7 ± 2	11.1 ± 2.2	0.139
Hematocrit (%) mean ± SD	36.1 ± 5.6	34.6 ± 5.3	0.183
C‐reactive protein (mg/L) mean ± SD	27.8 ± 31.8	21.1 ± 33.8	0.032^a^
ESR (mm/h) mean ± SD	44 ± 34.4	37.4 ± 29.5	0.289
Albumin (g/dL) mean ± SD	3.7 ± 0.8	3.8 ± 0.8	0.480
25(OH)D_3_ (ng/mL) mean ± SD	22.1 ± 5.7	28.2 ± 16.8	0.008^e^
Calprotectin >250 µg/dL (%)	28 (90.3)	40 (75.5)	0.094
Treatment			
Immunosuppressive, (%)	35 (92%)	76 (89%)	0.753
Biologic, (%)	38 (97.4%)	54 (75%)	0.003^c^
Biologic (optimization, 2nd biologic), (%)	12 (31.6)	18 (21.2)	0.004^c^
Surgery, (%) one or more	17 (43)	20 (23)	0.036^b^
Death	1	—	

Abbreviations: 25(OH)D_3_, 25‐hydroxyvitamin D_3_; ESR, erythrocyte sedimentation rate; PCDAI, pediatric Crohn′s disease activity index; SD, standard deviation; U‐SBCD, upper and small bowel Crohn′s disease.

^a^ Mann–Whitney's test.

^b^ Chi‐square test.

^c^ Likelihood ratio test.

^d^ Analysis of variance.

^e^ Student *t*‐test.

Diagnostic delay exceeded 1 year in both groups, with a more pronounced trend in U‐SBCD (17 ± 15.5 months vs. 13 ± 10.9 months; *p* = 0.191), though not statistically significant. Disease activity assessed by PCDAI revealed significantly higher rates of moderate‐to‐severe disease in U‐SBCD (66% vs. 32%; *p* < 0.001).

#### 3.2.1 Nutritional status and growth impairment

U‐SBCD patients exhibited significantly greater height impairment (mean height 156 ± 20.8 cm vs. 143.3 ± 26.1 cm; *p* = 0.010) and lower BMI/age *z*‐scores (−1.14 ± 1.57 vs. −0.44 ± 1.62; *p* = 0.029) compared to non‐SBCD. While these *z*‐scores did not reach severe malnutrition threshold (*z* < −2), they represent clinically relevant nutritional deficits preceding overt growth failure.

#### 3.2.2 Inflammatory markers

Inflammatory markers showed significantly elevated CRP in U‐SBCD (27.8 ± 31.8 mg/L vs. 21.1 ± 33.8 mg/L; *p* = 0.032), while ESR did not differ (44 ± 34.4 mm/h vs. 37.4 ± 29.5 mm/h; *p* = 0.289). FC was elevated (>250 μg/g) in majority of patients in both groups (90.3% in U‐SBCD vs. 75.5% in non‐SBCD; *p* = 0.094), but did not differentiate phenotypes.

#### 3.2.3 Disease behavior and complications

U‐SBCD showed higher proportions of stricturing (B2:23.7% vs. 7.1%) and penetrating (B3: 10.5% vs. 16.5%) complications compared to inflammatory behavior (B1: 65.8% vs.76.4%; *p* = 0.050).

Perianal disease (p modifier) was significantly more common in non‐SBCD (36.5%) compared to U‐SBCD (18.4%; *p* = 0.045). Extraintestinal manifestations occurred similarly in both groups (21% vs. 23%).

Serum 25‐OH vitamin D₃ levels were significantly lower in U‐SBCD compared with non‐SBCD (22.1 ± 5.7 ng/mL vs. 28.2 ± 16.8 ng/mL; *p* = 0.008). Albumin levels showed a non‐significant trend toward lower values in U‐SBCD (3.7 ± 0.8 g/dL vs. 3.8 ± 0.8 g/dL; *p* = 0.480).

#### 3.2.4 Treatment

Instead of EEN, patients received parenteral nutrition, which was provided exclusively during the preoperative hospitalization. The majority of patients initiated treatment with corticosteroids and immunosuppressive therapy (azathioprine/methotrexate), regardless of phenotype (92% U‐SBCD vs. 89% non‐SBCD; *p* = 0.753). However, the use of biologic therapy was significantly higher in the U‐SBCD group (97.4% vs. 75%; *p* = 0.003).

Treatment optimization or escalation to second‐line biologics was required more frequently in U‐SBCD, reflecting inadequately controlled disease activity due to non‐response, loss of response, intolerance, or steroid dependence. Consequently, nearly one‐third of U‐SBCD patients showed suboptimal treatment responses, failing to achieve mucosal healing and often progressing to complicated disease behavior.

#### 3.2.5 Surgical outcomes

**Figure 1:**
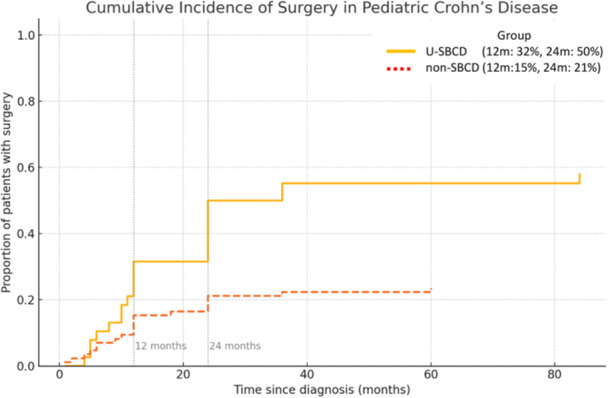
Kaplan–Meier curves demonstrating a significantly higher cumulative incidence of surgery in U‐SBCD over the 18‐month follow‐up period U‐SBCD non‐SBCD (ileum, colon, ileocolonic Crohn′s disease). U‐SBCD, upper and small bowel Crohn′s disease.

Patients with U‐SBCD more frequently presented with obstruction requiring emergency surgery. Stricture‐related surgery was significantly more common in U‐SBCD, with a higher subset undergoing emergency procedures (43% vs. 23%; *p* = 0.036). Multivariate logistic regression revealed U‐SBCD patients had threefold increased likelihood of surgery (odds ratio [OR] 3.02; 95% confidence interval [CI]: 1.02–8.94; *p* = 0.046), independent of other variables (Table 3 and Figure 1). A female child 9‐years old in U‐SBCD group died from sepsis following intestinal perforation during second surgical procedure.

**Table 3:** Multivariate logistic regression model to demonstrates the association between clinical characteristics and the occurrence of surgeries in U‐SBCD.

Variable	Odds ratio	95% Confidence interval		*p*
		Lower	Upper	
Years of delay diagnosis	1.22	1.06	1.39	0.004*
Weight loose (*z*‐score < −2)	8.30	2.17	31.66	0.002*
Abdominal pain	4.54	1.59	12.95	0.005*
Growth	0.33	0.10	1.04	0.059
Albumin	1.85	0.98	3.52	0.059
25(OH) vitaminD_3_	0.95	0.90	1.01	0.082
C‐reactive protein	1.00	0.99	1.02	0.637
Surgeries	3.02	1.02	8.94	0.046*
Stricturing behavior (B2 vs. B1)	2.50	1.30	4.81	0.006*
Penetrating behavior (B3 vs. B1)	4.10	2.05	8.20	<0.001*

Abbreviation: USCD, upper and small bowel Crohn's disease

* *p* ≤ 0.05.

## 4 DISCUSSION

This multicenter Brazilian study represents the first comprehensive characterization of pediatric U‐SBCD in Latin America, addressing a critical gap in LMIC literature. Our findings demonstrate that children and adolescents in Brazil with U‐SBCD present with distinctly aggressive phenotype compared to non‐SBCD, characterized by greater growth impairment, nutritional deficits, reduced treatment responsiveness, and threefold increased surgical risk. These observations align with reports from high‐income countries but emerge within a healthcare context where IBD remains poorly integrated into medical education and primary care training, potentially exacerbating diagnostic delays and adverse outcomes.^7^, ^23^

The 17‐month median diagnostic delay in our U‐SBCD cohort, though not statistically different from non‐SBCD, carried significant consequences. Each additional year of delay increased U‐SBCD complication odds by 22%, consistent with Ricciuto et al.′s report that delays exceeding 10.8 months confer 2.5‐fold increased stricturing or penetrating disease risk.^12^ The Kaplan–Meier curve (Figure 1) demonstrates persistently higher surgical rates in U‐SBCD patients throughout follow‐up, illustrating this temporal relationship. The later age at diagnosis observed in U‐SBCD versus no‐SBCD could be attributed to pediatric gut‐specific developmental and immunological factors.^8^

Our multivariate analysis identified a discriminative clinical pattern with important practical implications: chronic abdominal pain combined with significant weight loss (*z*‐score < −2) conferred 4.45‐ and 8.3‐fold increased odds of U‐SBCD, respectively. These clinical signs should trigger immediate suspicion for proximal small bowel involvement, prompting early cross‐sectional imaging rather than relying solely on normal ileocolonoscopy. In particular, diarrhea, often considered a hallmark IBD symptom, showed no discriminative value between phenotypes, underscoring atypical U‐SBCD presentation where symptoms may be subtle or intermittent, easily dismissed as functional disorders or infection.^2^, ^16^

In Brazil, as in many LMICs, IBD remains absent from primary care training programs, resulting in limited practitioner awareness. This educational gap represents a modifiable risk factor demanding immediate attention. Educational initiatives targeting general pediatricians and integration of validated screening tools such as IBD‐REFER criteria could significantly reduce diagnostic delays.^24^ IBD‐Associations for clinicians, parents, and patients contribute to earlier recognition and prevention of avoidable morbidity.

Our data demonstrate nutritional compromise manifests early in U‐SBCD, with BMI *z*‐scores declining temporally linked to symptom onset. The significantly lower BMI/age *z*‐scores in U‐SBCD, while not reaching severe malnutrition threshold (*z* < −2), represent clinically meaningful deficits warranting intervention. These findings suggest weight loss may serve as an earlier and more sensitive nutritional alarm than linear growth failure in U‐SBCD, enabling clinicians to identify high‐risk patients before overt malnutrition develops. The 42% prevalence of growth impairment in our U‐SBCD patients demonstrates both duration of unrecognized disease and inflammatory burden from extensive small bowel involvement. Additionally, significantly lower 25‐OH vitamin D₃ levels in U‐SBCD highlight malabsorptive consequences of proximal small bowel inflammation, which may further compromise bone health and immune function.^25^ These nutritional deficits underscore need for proactive nutritional assessment and supplementation as integral components of U‐SBCD management, rather than reactive interventions after malnutrition is established.

While FC remains gold‐standard noninvasive biomarker for pediatric CD, our data confirm its limited utility in differentiating U‐SBCD from non‐SBCD phenotypes. This finding aligns with emerging literature demonstrating reduced FC sensitivity for isolated small bowel disease, likely reflecting the biomarker′s dependence on colonic inflammation and stool transit through diseased segments.^26^ The high prevalence (90.3%) of markedly elevated FC (>250 μg/g) at diagnosis in U‐SBCD patients indicates substantial inflammatory burden; however, in endemic areas, parasitic infections can also elevate FC, potentially delaying definitive imaging and endoscopic evaluation.^26^

Cross‐sectional imaging, MRE/CTE remains essential in evaluation of suspected U‐SBCD, particularly given biomarker limitations or when ileocolonoscopy may miss small bowel involvement if the terminal ileum appears normal. In our cohort, all patients underwent MRE/CTE in accordance with ESPGHAN recommendations, allowing full‐thickness bowel wall assessment and reliable detection of strictures, penetrating complications, and extramural disease not accessible to ileocolonoscopy.^16^ IUS, though unavailable at our centers during study period, represents a promising emerging tool for noninvasive monitoring of small bowel disease activity.^27^ IUS is an emerging diagnostic tool in Brazil; the number of trained professionals remains limited.

The significantly higher biologic therapy utilization in U‐SBCD reflects disease severity and inadequacy of conventional immunosuppression. Critically, 31.6% of U‐SBCD patients required treatment optimization or escalation to second‐line biologics, indicating primary or secondary loss of response. This suboptimal treatment response may reflect higher biologic concentrations needed to achieve small bowel mucosal healing compared to colonic disease; delayed diagnosis resulting in established fibrotic strictures less responsive to therapy; and pharmacologically challenging drug dynamics in proximal small bowel.^8^, ^28^ The off‐label use of vedolizumab in refractory cases highlights limited therapeutic armamentarium available for pediatric CD in Brazil. Currently, only infliximab and adalimumab are approved for pediatric use.

In Brazil, significant barriers limit optimal U‐SBCD management. EEN, internationally recommended as first‐line induction therapy, remains underutilized due to prohibitive costs (often exceeding biologics), inconsistent reimbursement, and poor tolerability requiring inpatient initiation.^28^, ^29^ Consequently, even among patients with severe malnutrition, EEN was rarely used; instead, parenteral nutrition was provided only during preoperative hospitalization. Corticosteroids were predominant induction therapy despite well‐recognized adverse effects, particularly in children with growth impairment. Therapeutic options for anti‐TNF failure remain severely limited, and therapeutic drug monitoring requires sending samples outside Brazil, imposing substantial logistical and financial barriers to treatment optimization.

These resource limitations underscore importance of treat‐to‐target strategies with proactive clinical and biochemical monitoring using widely available biomarkers (CRP, albumin, FC when accessible) to guide treatment intensification, rather than reactive escalation after clinical failure.^19^ We echo calls for expedited approval of additional biologics, including vedolizumab, ustekinumab, and emerging agents, for critically ill children to prevent lifelong sequelae.^22^, ^28^

Surgical intervention occurred in 43% of U‐SBCD patients versus 23% of non‐SBCD (*p* = 0.036), with multivariate analysis confirming U‐SBCD as independent 3.02‐fold risk factor after adjusting for confounders. The Kaplan–Meier curve (Figure 1) demonstrates this surgical divergence emerges early and persists throughout follow‐up. Many U‐SBCD patients presented acutely with obstructive symptoms requiring emergency intervention, reflecting advanced disease at diagnosis. Surgical approaches included strictureplasty (to preserve bowel length), segmental resection, stoma formation, and Kono‐S anastomosis (to reduce postoperative recurrence).^30^ Paradoxically, perianal disease was significantly less common in U‐SBCD than non‐SBCD, contrary to general association between perianal involvement and severe CD. This observation aligns with previous reports suggesting perianal disease may be more closely linked to colonic/ileocolonic phenotypes than small bowel disease,^5^ possibly reflecting differences in lymphatic drainage or microbial exposure.

The death of a 9‐year‐old girl from complications following intestinal perforation during second surgical procedure tragically illustrates the life‐threatening potential of unrecognized U‐SBCD and delayed referral. Although uncommon, this outcome underscores profound clinical impact of missed or late diagnosis and emphasizes urgent need for enhanced IBD awareness. The 30% prevalence of depressive or anxiety symptoms requiring psychiatric intervention, identical across both phenotypes, highlights substantial psychological burden of pediatric CD. This finding emphasizes need for integrated multidisciplinary care models that incorporate mental health screening.^6^

Brazil is a country with continental dimensions, marked sociodemographic heterogeneity, and extensive admixture of European, African, Asian, Indigenous, and Middle Eastern ancestries, creating a distinctive population for IBD research. The observation that ethnically diverse phenotypic patterns in Brazil′s, predominantly African‐admixed population, more closely resemble Asian than Caucasian cohorts raises important questions about biological mechanisms of disease expression.^31^

This multicenter study provides valuable data on SBCD across diverse Brazilian ancestry groups, contributing to the global PIBD registry. However, the retrospective design introduces potential selection bias and data variability across centers. While 18‐month follow‐up period is sufficient to capture early surgical outcomes, limits assessment of long‐term disease evolution and postoperative recurrence.

## 5 CONCLUSION

This first large‐scale multicenter in Brazil demonstrates U‐SBCD presents as high‐risk phenotype characterized by diagnostic delays, growth impairment, poor treatment response, and increased surgical risk. Each year of diagnostic delay increases threefold higher surgical risk, underscoring delay as modifiable risk factor.

In LMIC settings where IBD remains poorly integrated into medical education, these findings highlight urgent need for systematic IBD training among primary care providers and pediatricians. Early diagnostic protocols and phenotype‐specific management strategies represent modifiable interventions that can transform U‐SBCD from life‐threatening emergency into manageable chronic condition, even in resource‐limited settings.

In genetically admixed populations, disease phenotypes can differ from traditionally studied Caucasian cohorts. The observation that phenotypic patterns in Brazil′s ethnically diverse, predominantly African‐admixed population more closely resembles Asian than Caucasian cohorts raises important questions about the underlying biological mechanisms of disease.

## 7 CONFLICT OF INTEREST STATEMENT

The authors declare no conflicts of interest.
